# Genomic characterization and cross-species transmission potential of hedgehog coronavirus

**DOI:** 10.1016/j.onehlt.2024.100940

**Published:** 2024-11-19

**Authors:** Andreia V.S. Cruz, Sérgio Santos-Silva, Luís Queirós-Reis, Clarisse Rodrigues, Vanessa Soeiro, Rachael E. Tarlinton, João R. Mesquita

**Affiliations:** a Instituto de Ciências Biomédicas Abel Salazar (ICBAS), Universidade do Porto, 4050-313 Porto, Portugal; b Centro de Recuperação e Interpretação do Ouriço, 4470-372 Maia, Portugal; c Centro de Recuperação de Fauna do Parque Biológico de Gaia, 4430-812 Vila Nova de Gaia, Portugal; d School of Veterinary Medicine and Science, University of Nottingham, Sutton Bonington Campus, Loughborough LE12 5RD, United Kingdom; e Epidemiology Research Unit (EPIUnit), Instituto de Saúde Pública da Universidade do Porto, 4050-600 Porto, Portugal; f Laboratório para a Investigação Integrativa e Translacional em Saúde Populacional (ITR), 4050-600 Porto, Portugal

**Keywords:** Coronavirus, Hedgehogs, Erinaceus coronavirus, Public health, Portugal

## Abstract

In the 21st century, three betacoronaviruses (SARS-CoV, MERS-CoV and SARS-CoV-2) have emerged in humans worldwide as a result of animal spillover, causing severe respiratory infections and resulting in more than seven million deaths. In 2013, a novel *Betacoronavirus* closely related to MERS-CoV (*Betacoronavirus cameli)* was discovered in European hedgehogs (*Erinaceus europaeus*), raising questions on the possibility of hedgehog-to-human transmission. Hence, the present study aimed to investigate and characterize the presence and genetic diversity of coronaviruses in hedgehogs from Portugal, as well as their potential for cross-species transmission. To achieve this, fecal samples from 110 hedgehogs at two recovery centers and one environmental non-governmental organization were tested for coronaviruses using a broad-spectrum nested RT-PCR assay targeting the *RdRp* gene. Of these samples, 24.5 % tested positive, most belonging to the *Betacoronavirus* genus. However, the present study also reports, for the first time, *Alphacoronaviruses* in hedgehogs, showing 100 % identity with a Bat coronavirus (a variant of *Alphacoronavirus miniopteri)*. The genome sequencing of one betacoronavirus-positive sample yielded 65 % of a full-length genome, with the closest homology (93.5 %) to *Betacoronavirus erinacei* from the United Kingdom. Computational protein-protein docking studies predicted the binding affinity between the spike protein of hedgehog coronavirus and cell receptors of mammal species that interact with hedgehogs. The results obtained raise the question of whether hedgehog CoV uses the same receptor as MERS-CoV or a different receptor to enter host cells. Thus, this study enhances our understanding of the epidemiology of coronaviruses, emphasizing the need for further investigation into cross-species transmission risks.

## Highlights

• 24.5 % of hedgehogs tested positive for coronaviruses in Portugal.
• First report of Alphacoronaviruses detected in hedgehogs.
• Docking studies suggest hedgehog CoV may not use the same cell receptor as MERS-CoV.

## 1 Introduction

Coronaviruses (order *Nidovirales*, family *Coronaviridae*, subfamily *Coronavirinae*) are a group of enveloped, positive-sense single-stranded RNA viruses [1,2]. The subfamily *Coronavirinae* is comprised of four genera: *Alpha*-, *Beta*-, *Gamma*-, and *Deltacoronavirus*. While gamma- and deltacoronaviruses primarily infect birds, alpha- and betacoronaviruses infect various mammalian species, including humans [2]. They are widespread and responsible for respiratory, enteric, hepatic, and neurological diseases with variable severity [3].

In the 21st century, three betacoronavirus have emerged worldwide, leading to severe respiratory infections in humans [4]. In November 2002, the severe acute respiratory syndrome coronavirus (SARS-CoV) (*Betacoronavirus pandemicum)* emerged in Guangdong province, China, with an origin in bats and transmitted to humans *via* civets [5,6]. In 2012, a novel coronavirus, later named Middle East respiratory syndrome coronavirus (MERS-CoV) (*Betacoronavirus cameli)*, was isolated from a man with pneumonia in Saudi Arabia. Although it had been suggested that the virus originated in bats, MERS-CoV is currently an endemic virus of dromedaries (*Camelus dromedarius*) in the Middle East, serving as the source of transmission to humans [[7], [8], [9]]. In late December 2019, several cases of patients with pneumonia were reported linked to a seafood and wet animal wholesale market in Wuhan, Hubei Province, China. The new pandemic, designated coronavirus disease 2019 (COVID-19), was caused by the SARS-CoV2 virus (*B. pandemicum)* [10].

Given the considerable surge in newly discovered bat coronaviruses subsequent to the SARS outbreak, particularly in insectivorous bats, it was hypothesized that other insectivorous mammals could also host coronaviruses [11]. This could particularly relate to the Eulipotyphla animal order, which includes hedgehogs, which are phylogenetically related to the order Chiroptera [11]. In 2013, a novel *Betacoronavirus* was discovered in European hedgehogs (*Erinaceus europaeus*) in Germany (*Betacoronavirus erinacei)*. The Erinaceus coronavirus (EriCoV), a *B. erinacei* previously classified as *Hedgehog coronavirus 1*, groups phylogenetically within clade C betacoronaviruses in close relationship to MERS-CoV and bat coronaviruses [11]. To date, two hedgehog coronaviruses have been identified: EriCoV was reported in European hedgehogs in France, Great Britain, Italy, and Poland [[12], [13], [14], [15]], while HKU31 was detected in Amur hedgehogs (*Erinaceus amurensis*) in China [16,17].

European hedgehogs are widespread in Europe and have become synanthropic animals [18]. The conservation status of the European hedgehog has declined, now being classified as near threatened globally and for the European Union [19]. Factors such as agricultural intensification along with urban development, have likely contributed to the loss and degradation of essential nesting and foraging habitats for this species [19]. The growing number of encounters between hedgehogs and humans have sparked concerns about the potential spread of zoonotic viruses, particularly coronaviruses, and their spillover from wildlife species to humans and domestic animals [20].

Cell infection and disease is initiated by the interaction between the coronavirus spike protein and a receptor on the host cell's surface, essential to allow the virus to enter the cell [21]. In particular, interaction occurs between a cell receptor and the coronavirus spike protein receptor-binding domain (RBD) located within the S_1_ subunit [22,23]. Among the four well-characterized human coronavirus receptors, three are transmembrane proteases – angiotensin-converting enzyme 2 (ACE2), dipeptidyl peptidase 4 (DPP4), and aminopeptidase N (APN) – while the fourth is carcinoembryonic antigen-related cell adhesion molecule 1 (CEACAM1). For instance, ACE2 is widely used by sarbecoviruses, betacoronavirus lineage B (such as SARS-CoV-2), and for NL63, an alphacoronavirus. APN mediates the entry receptor of several alphacoronaviruses and a deltacoronavirus. In contrast, DPP4 is recognized as an entry receptor exclusively for certain merbecoviruses (*Betacoronavirus* lineage C), such as MERS-CoV, HKU4 (T*ylonycteris bat coronavirus)* and HKU25 (*Pipistrellus bat coronavirus)* [23] while the carcinoembryonic antigen-related cell adhesion molecule 1 (CEACAM1) is recognized by mouse hepatitis coronavirus (MHV) (*Betacoronavirus muris)*, and Bovine coronavirus (BCoV) (*Alphacoronavirus chicagoense)* [24]. The binding ability of the coronavirus RBD to its host receptor is a determining factor in defining cell tropism, host range of coronaviruses and cross-species infection [25]. Previous structural analysis of the Italian EriCoV RBD and HKU31 RBD suggested that these hedgehog-derived coronaviruses might not bind to human receptors [[15], [16], [17]]. However, this alone does not preclude cross-species transmission, as some coronaviruses can utilize alternative co-receptors for cell entry [15].

The primary objective of this study was to investigate and characterize the diversity of coronaviruses present in hedgehogs from Portugal using PCR-based techniques and full genome sequencing. Then, computational protein docking studies were employed to predict the binding affinity between the spike protein of hedgehog CoV and the DPP4 receptor of mammal species that closely interact with hedgehogs to predict infection. By achieving these objectives, we hope to contribute to a better understanding of the epidemiology and ecology of coronaviruses in hedgehogs, ultimately aiding in the development of strategies for disease surveillance and management.

## 2 Materials and methods

### 2.1 Sample collection

Fecal samples from 110 hedgehogs were obtained from two recovery centers and one environmental non-governmental organization in Portugal. These hedgehog rescue centers specialize in evaluating and treating animals found in compromised conditions, including those that are debilitated, injured (particularly victims of road traffic accidents), orphaned, or otherwise distressed. After receiving treatment for their initial condition, the animals remain at the facilities in specialized rehabilitation enclosures where they can fully regain their physical strength and fitness. During this rehabilitation period, staff carefully monitor the animals to ensure they demonstrate the necessary survival behaviors and instincts characteristic of wild hedgehogs. Only after successfully completing all these rehabilitation protocols and demonstrating appropriate wild behaviors are the animals deemed suitable for release back into their natural habitat. The animals are not collected for screening purposes.

**Fig. 1:**
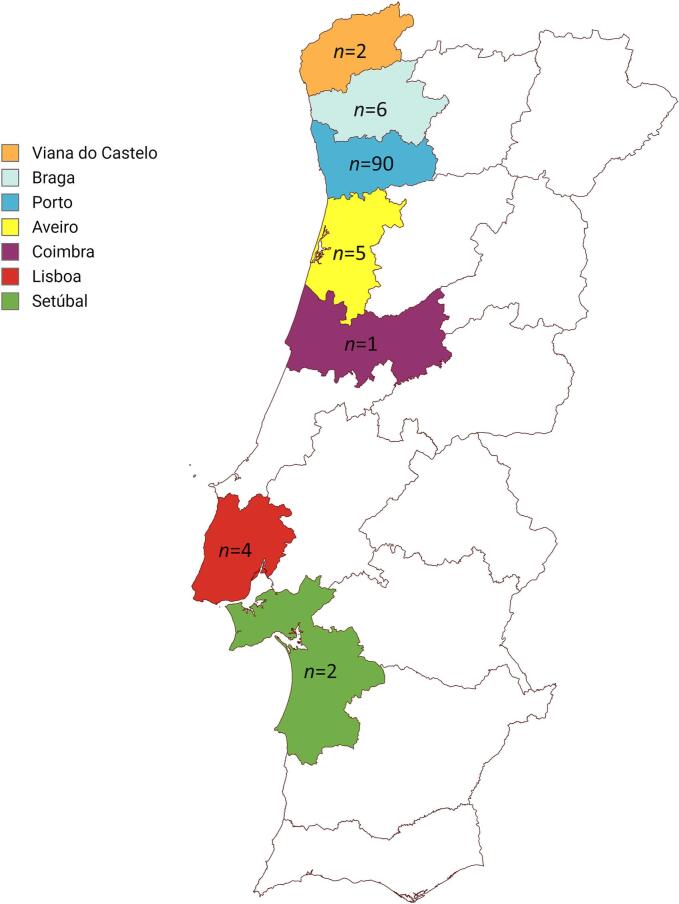
Geographical location, by municipalities, of the hedgehog capture sites in Portugal.

Thirty stool samples of European hedgehogs (*E. europaeus*), collected between September 2021 and April 2023, were provided by Centro de Recuperação de Fauna do Parque Biológico de Gaia (CRF-PBG). Additionally, 76 fecal samples from European hedgehogs were collected in Centro de Recuperação e Interpretação do Ouriço (CRIDO), between December 2022 and May 2023. Stool samples from three African pygmy hedgehog (*Atelerix albiventris*) and one long-eared hedgehog (*Hemiechinus auritus*), collected in May 2023, were provided by the Associação Amigos Picudos. While the *E. europaeus* were collected from the wild, the non-native species were found in public areas and taken to the Associação Amigos Picudos for care. The sale of non-native hedgehog species, such as African pygmy hedgehogs and long-eared hedgehogs, is permitted in Portugal, indicating that these animals were likely kept as pets and abandoned. Among these animals, 51 were males, 44 were females, and the sex of 15 individuals was not provided at the time of collection and could not be determined after the release of the corresponding healthy animals. Samples provided by CRF-PBG (*n* = 30) originated from animals found in the Porto (*n* = 28), Viana do Castelo (*n* = 1) and Aveiro (*n* = 1) districts (Fig. 1). Regarding the samples from CRIDO, the majority were also from hedgehogs found in the Porto district (*n* = 61), with the remaining collected from animals captured in Braga (*n* = 6), Lisboa (*n* = 3), Setúbal (*n* = 1), Aveiro (*n* = 4), and Viana do Castelo (*n* = 1) (Fig. 1). The animals sampled from the Associação Amigos Picudos were from Coimbra (n = 1), Setúbal (n = 1), Porto (n = 1) and Lisboa (n = 1).

Individual fecal samples were passively collected from the cages where the hedgehogs were individually housed. The cages are cleaned daily with bedding changes and properly sanitized between occupants, reducing the possibility of cross-contamination. No animals in this study showed signs of gastrointestinal disease. Fresh samples were immediately refrigerated and transported within a maximum of two hours to the laboratory, where they were stored at −20 °C until RNA extraction, which was completed within two weeks after collection.

### 2.2 RNA extraction

Fecal suspensions (10 %) were prepared in phosphate-buffered saline (pH 7.2) and centrifuged at 8000 ×*g* for 5 min. A total of 140 μL of each clarified supernatant was used for the RNA extraction with the QIAamp viral RNA mini kit (Qiagen, Hilden, Germany), following the manufacturer's instructions, and the QIAcube® automated platform (Qiagen, Hilden, Germany). The RNA was eluted in RNase-free water and stored at −80 °C until further use.

### 2.3 Molecular detection of coronaviruses

For the first screening, a broad-spectrum pan-coronavirus nested RT-PCR assay was performed targeting the highly conserved RNA-dependent RNA polymerase (*RdRp*) gene. This protocol has been demonstrated to be the most sensitive for detecting both alpha- and betacoronaviruses [26].

For the first round of PCR, the Xpert One-Step RT-PCR kit (GRiSP®, Porto, Portugal) was used in combination with the primer set Hu-F/Hu-R to amplify a 668 bp fragment [27] (Table 1). The cycling conditions included the cDNA synthesis at 45 °C for 15 min, an initial denaturation at 95 °C for 3 min, followed by 40 cycles of denaturation at 95 °C for 15 s, annealing at 52 °C for 15 s, and extension at 72 °C for 2 s and a final extension at 72 °C for 10 min. The PCR reaction for the second round was performed using Xpert Fast Hotstart Mastermix 2× with dye (GRiSP®, Porto, Portugal) and the primer set Poon-F/Chu06-R1 to amplify a 440 bp fragment [3,28]. The cycling conditions were identical to the first round, without cDNA synthesis and adjusting the annealing temperature to 50 °C. Positive (GenBank accession number OQ613363) and negative (RNase-free water) controls were tested simultaneously with the other samples, using the same conditions. PCR products were electrophoresed on 1 % agarose gels stained with Xpert Green Safe DNA gel dye (GRiSP®, Porto, Portugal) at 120 V for 30 min and visualized under ultraviolet light. Molecular weights were estimated by comparison with GRS Ladder 100 bp (GRiSP®, Porto, Portugal).

**Table 1:** Oligonucleotides used for the molecular identification and characterization of coronaviruses.

Oligonucleotide	Sequence (5′–3′)	Reference
Hu-F	AARTTYTAYGGHGGYTGG	[27]
Hu-R	GARCARAATTCATGHGGDCC	
Poon-F	GGTTGGGACTATCCTAAGTGTGA	[3]
Chu06-R1	CCATCATCAGATAGAATCATCAT	[28]

### 2.4 Sanger sequencing and phylogenetic analysis of partial *RdRp* sequences

Amplicons with the expected size (440 bp) were purified using the GRS PCR & Gel Band Purification Kit (GRiSP®, Porto, Portugal) and bidirectionally sequenced by Sanger dideoxy sequencing with the primers Poon-F and Chu06-R1.

Nucleotide sequences were edited, aligned by ClustalW, and analyzed using the BioEdit Sequence Alignment Editor version 7.2.5. The obtained consensus sequences were compared with the available sequences in the NCBI database GenBank, using the Nucleotide Basic Local Alignment Search Tool.

The software MEGA version X [29] was used for the phylogenetic analysis and the Interactive Tree Of Life (iTOL) platform [30] for editing. The analysis included the sequences obtained in this study and representative alpha, beta, gamma, and delta coronavirus sequences obtained from GenBank and was performed using the maximum-likelihood (ML) approach. The General Time Reversible model was determined by MEGA version X to be the most effective replacement model, estimating the ML bootstrap values using 1000 replicates [29,31].

### 2.5 Statistical analysis

The prevalence of coronavirus was calculated using the proportion of positive samples from the total number of tested samples with a 95 % confidence interval (95 % CI).

### 2.6 High-throughput sequencing and genome analyses

All samples that tested positive for the coronavirus *RdRp* gene were assessed for purity, and five samples meeting the recommended ratios were sent for RNA sequencing by Novogene (Cambridge, United Kingdom), utilizing the Illumina NovaSeq 6000 platform. FastP v0.23.1 was employed for quality filtering and trimming to eliminate adapters, low-quality reads and duplicates [32]. The taxonomic classification of paired-end reads was performed using the Kraken2 viral Refseq database (retrieved on 24 July 2023) of Kraken v2.1.2 [33]. Reads were assembled with the rnaviralSPAdes module within the SPAdes genome assembler v3.15.4 [34], employing default parameters. Contigs were queried against the Nucleotide BLAST database from NCBI (retrieved on 15 November 2023). The assembled contigs identified as coronavirus sequences were indexed and extracted for subsequent analysis using the SAMtools v1.20 index option [35]. The assembled genome was annotated in Geneious Prime 2024.0 using the NCBI coronavirus reference sequence for *B. erinacei* (Taxonomy ID: 1965093).

### 2.7 Phylogenetic analysis of RBD amino acid region of spike glycoprotein

The phylogenetic analysis of the full RBD region was conducted using the software MEGA version X [29]. The analysis included the RBD amino acid sequence obtained in this study and RBD sequences of SARS-CoV (AAP41037), MERS-CoV (YP_009047204), SARS-CoV2 (YP_009724390) and *B. erinacei* (QCC20713) obtained from GenBank. The spike protein of NL63 (AFV53148) was used as outgroup. The evolutionary history was inferred by using the Maximum Likelihood (ML) method and Whelan and Goldman + Freq. model [29,36] in MEGA X, estimating the ML bootstrap values using 1000 replicates. The Interactive Tree Of Life (iTOL) platform [30] was used for editing.

### 2.8 Homology modeling and protein-protein docking simulations

The crystal structure of human DPP4 complexed with MERS-CoV RBD was downloaded from the Protein Data Bank (PDB) [37] (http://www.rcsb.org/, accessed on January 12, 2024; PDB ID 4L72). In the absence of structure models of hedgehog DPP4 and its major predators in Europe, the respective DPP4 3D models were constructed through homology modeling using SWISS-MODEL [38] (https://swissmodel.expasy.org/), employing human DPP4 (PDB ID: 2QT9) as the template. For this purpose, the amino acid sequences of the DPP4 proteins of the dog (ID: A0A8C0NCU9), cat (ID: Q9N2I7), and red fox (ID: A0A3Q7RX66), were retrieved from UniProt [39] (https://www.uniprot.org/, accessed on April 17, 2024) and the amino acid sequences of hedgehog DPP4 (ID: XP_060033795XM_060177812) and Eurasian badger DPP4 (ID: XP_045874346) were retrieved from GenBank (https://www.ncbi.nlm.nih.gov/genbank/, accessed on April 26, 2024).

Similarly, the crystal structures of human (PDB ID: 6M1D), dog (PDB ID: 7E3J), cat (PDB ID: 7C8D), and fox (PDB ID: 8XYZ) ACE2 were downloaded from the PDB (accessed on August 2, 2024). The amino acid sequences of hedgehog ACE2 (GenBank ID: XP_060038995) and Eurasian badger ACE2 (GenBank ID: XP_045850934) were retrieved from GenBank (accessed on July 29, 2024), and their respective 3D models were constructed through homology modeling using SWISS-MODEL, employing dog ACE2 (PDB ID: 7E3J) as the template.

An additional homology modeling of the hedgehog spike protein obtained here through full genome sequencing was built using SWISS-PROT, with the crystal structure PDB ID: 7U6R as the template.

The proteins were preprocessed with the Minimize Structure Chimera tool, version 1.17.3, and saved in PDB format.

Protein-protein docking studies between spike and DPP4 or ACE2 proteins were performed in triplicate using Haddock 2.4 [40,41], keeping the default parameters. Active residues were defined to provide insights into interacting residues [42,43]. The passive residues were automatically determined based on the specified active residues, encompassing all residues located on the interface surface and within a 6.5 Å radius of any active residue. The cluster models with the best HADDOCK score were saved in PDB format.

## 3 Results

### 3.1 Molecular detection and phylogenetic analysis

From the total 110 hedgehog fecal samples tested, 27 were found to be positive for coronavirus, representing an overall occurrence of 24.5 % (95 % CI: 16.8–33.7). All the positive samples belong to *E. europaeus*. The geographic origins of the positive animals are displayed in Table 2.

**Table 2:** Geographic origins of the coronavirus-positive hedgehogs identified in this study.

District	Nr of animals sampled	Nr of positives	Percentage of positives
Viana do Castelo	2	0	0
Braga	6	0	0
Porto	90	24	26.7
Aveiro	5	2	40
Coimbra	1	0	0
Lisboa	4	0	0
Setúbal	2	1	50
Total	110	27	24.5

BLAST analysis revealed that 25 of the sequences obtained shared between 96 % to 97 % identity with *B. erinacei* isolated from a *E. europaeus* in Germany in 2012 (NC_039207), and between 94.6 % to 96.2 % identity with *B. erinacei* sequence from a *E. europaeus* in the United Kingdom in 2014 (MK679660). The nucleotide homology among the 25 *B. erinacei* sequences in this study ranged from 96.67 % to 100 %. Additionally, two sequences exhibited 100 % identity with a Bat coronavirus, a variant of *Alphacoronavirus miniopteri* obtained from a *Miniopterus schreibersii* in Portugal in 2022 (OQ613364).

**Fig. 2:**
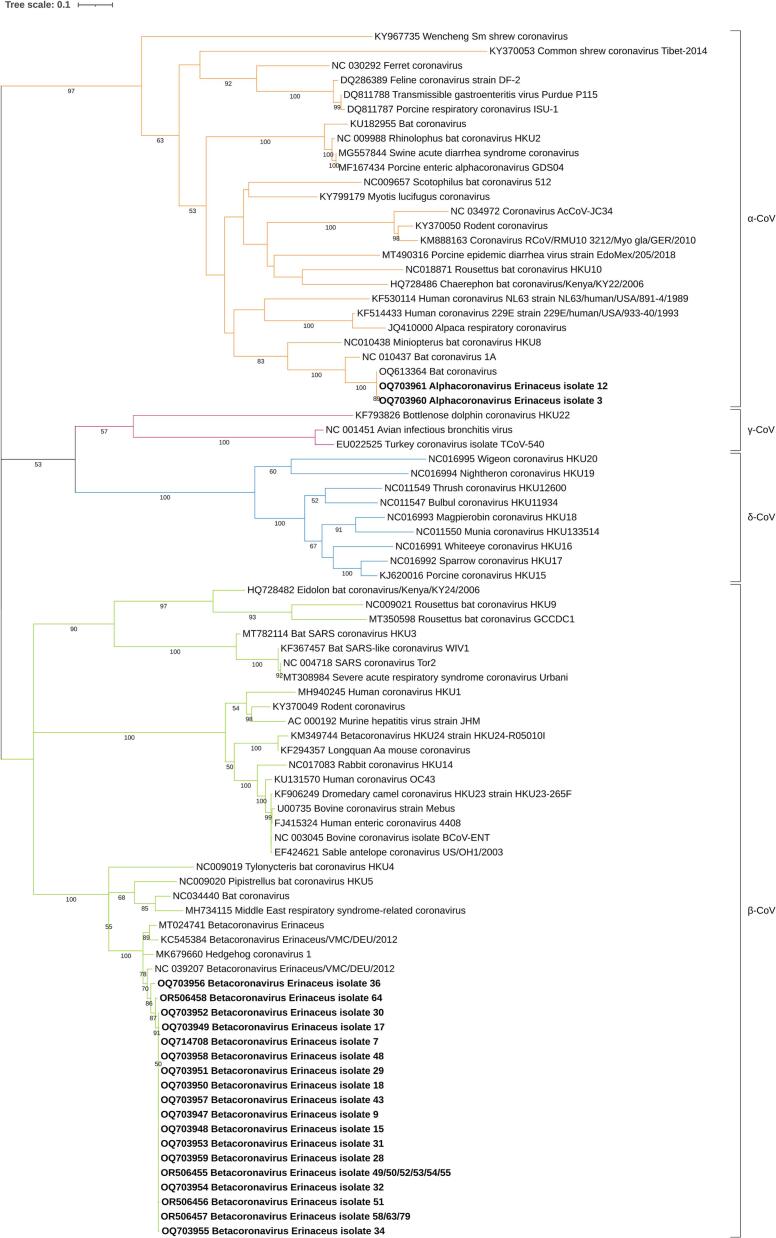
Phylogenetic analysis of the *RdRp* coronavirus nucleotidic sequences (403 bp) identified in this study (in bold) and reference sequences retrieved from GenBank. Genetic distances were calculated using the General Time Reversible model. Sequences are named with GenBank accession number and name of virus. Orange branches correspond to *Alphacoronavirus*, green to *Betacoronavirus*, pink to *Gammacoronavirus* and blue to *Deltacoronavirus*. Bootstrap values equal to or greater than 50 % are represented at the nodes. (For interpretation of the references to colour in this figure legend, the reader is referred to the web version of this article.)

Subsequent phylogenetic analysis confirmed that twenty-five positive sequences belonged to the *Betacoronavirus* genus, previously isolated from hedgehogs, and showed a close relationship to MERS-CoV-related viruses. In contrast, two sequences belonged to the *Alphacoronavirus* genus (Fig. 2).

The partial *RdRp* sequences obtained in this study were deposited in GenBank under accession numbers: OQ703947-OQ703961, OQ714708 and OR506455-OR506458.

### 3.2 Illumina sequencing taxonomic classification and genome organization

Of the five samples sent for RNA sequencing, only one exhibited sufficient quality to undergo sequencing. Taxonomic classification enabled the classification of reads as belonging to the Coronaviridae family. *De novo* assembly of the hedgehog sample yielded two coronavirus contigs of 18,379 kb and 8213 kb (G + C content of approximately 36.6 %) corresponding to 65 % of a full-length genome with 93.5 % homology to *B. erinacei* (MK679660) identified in an *E. europaeus* in the United Kingdom in 2014. The obtained partial genome was deposited in GenBank under accession number PP724614.

**Fig. 3:**
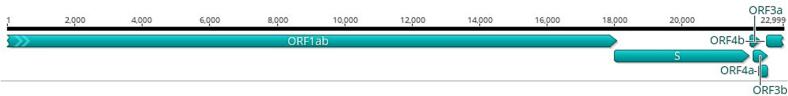
Genome organization of the partial genome derived from this study.

The hedgehog CoV partially sequenced in this study exhibits a genome organization similar to other betacoronaviruses of subgenus *Merbecovirus* [15,44]. It was possible to successfully sequence 18,076 bp of ORFab (open reading frame ab), including ORFa, the gene encoding the spike protein (S), ORF3a, ORF3b, ORF4a, and part of ORF4b (Fig. 3; Table 3). However, the sequencing process did not succeed in covering the 5’-UTR, the initial part of ORFab, ORF5, the genes encoding the structural proteins envelope (E), membrane (M), and nucleocapsid (N), ORF8b, and the 3’-UTR.

**Table 3:** Localization and length of predicted gene sequences of the hedgehog CoV genome partially sequenced in this study.

Gene name	Location on the genome	Length
ORF1ab	<1–18,076	>18,076
ORFa	>1–9989	>9989
S	17,994-21,986	3993
ORF3a	22,004-22,321	318
ORF3b	22,098-22,520	423
ORF4a	22,275-22,520	246
ORF4b	22,510- > 22,999	>490

### 3.3 Computational analysis of affinity dynamics between spike proteins and DPP4 receptors

**Fig. 4:**
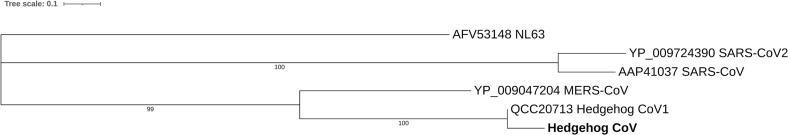
Phylogenetic tree generated for the RBD amino acid sequence obtained in the present study (in bold) and reference sequences retrieved from Genbank, using the Whelan and Goldman + Freq. model.

Despite the nucleotide similarity of the partial coronavirus genome obtained in the present study and MERS-CoV-related viruses, a phylogenetic analysis of its RBD region was conducted against the RBD region of the spike glycoprotein from coronaviruses associated with recent pandemics to better predict the receptor of this spike glycoprotein. As anticipated, the hedgehog CoV RBD from this study clusters with that of *B. erinacei*, and both cluster with MERS-CoV RBD (Fig. 4). Therefore, for the subsequent studies, we posited that the receptor for hedgehog CoV is likely DPP4, as for MERS-CoV [45].

**Fig. 5:**
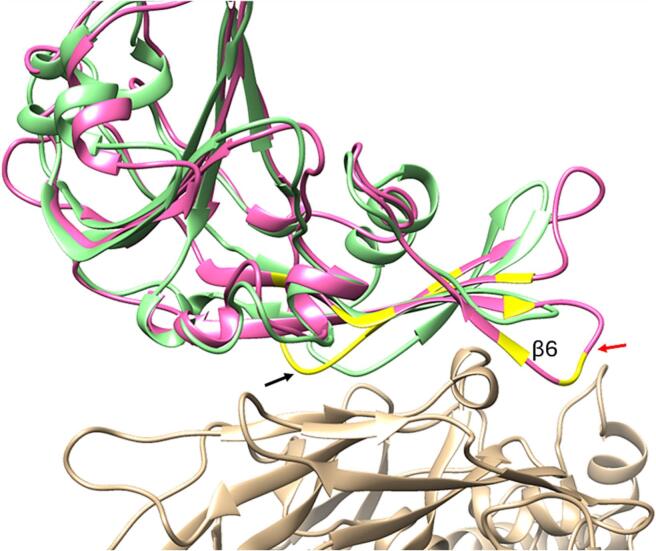
The model of the hedgehog CoV RBD model (light green) was superimposed onto the crystal structure of MERS-CoV RBD (pink) complexed with human DPP4 (beige) (PDB ID 4L72). The residues of MERS-CoV RBD known to contact with DPP4 are colored yellow (d < 3.6 Å). The β5-β6 linking loop of the MERS-CoV RBD is indicated with a red arrow and the β6-β7 linking loop is indicated with a black arrow. (For interpretation of the references to colour in this figure legend, the reader is referred to the web version of this article.)

Superimposing the structure model of the hedgehog CoV RBD with that of MERS-CoV RBD, the former lacks the short β6 strand and the β5-β6 linking loop which in MERS-CoV RBD contribute to the concave outer surface that accommodates a linker containing a short α-helix between blades 4 and 5 of DPP4 (Fig. 5). However, the hedgehog CoV RBD retains part of the concave surface and the C-terminal end of the long loop that, in MERS-CoV, connects the β6 and β7 strands and contacts blade 4 of DPP4, indicating a potential interaction with DPP4.

**Fig. 6:**
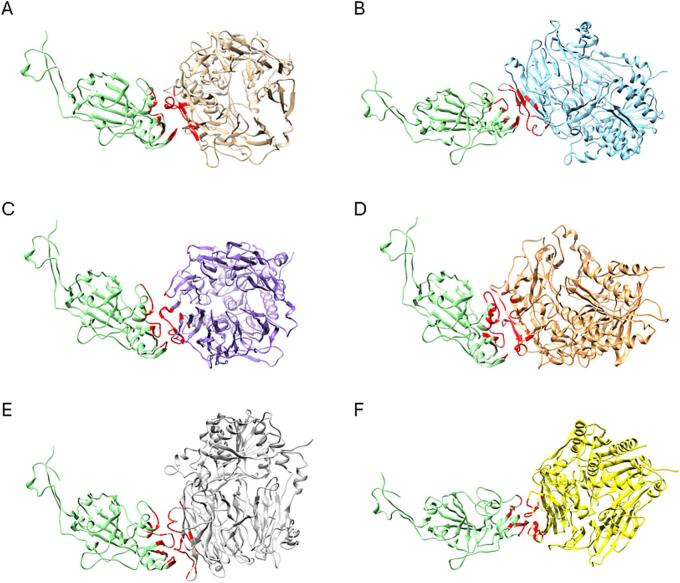
Models of structural interaction between the hedgehog CoV RBD (light green) and **(A)** human (beige), **(B)** dog (blue), **(C)** cat (purple), **(D)** fox (orange), **(E)** badger (grey) and **(F)** hedgehog (yellow) DPP4. The hedgehog CoV RBD residues that are within 5 Å of the contact surface with DPP4 are shown in red. (For interpretation of the references to colour in this figure legend, the reader is referred to the web version of this article.)

Protein docking simulations revealed that the binding of the hedgehog CoV spike protein was strongest with DPP4 of fox and cat, followed by badger DPP4, then with dog DPP4, and weakest with hedgehog and human DPP4 (Fig. 6, Table 4). To validate these findings, docking studies of MERS-CoV RBD with DPP4 from the same animal species were conducted. As anticipated, the MERS-CoV spike protein exhibited the most stable binding with human DPP4, followed by badger DPP4, and subsequently with hedgehog, fox, cat, and dog DPP4 (Table 4).

**Table 4:** HADDOCK scores obtained in the protein docking studies between the RBD of spike proteins from MERS-CoV and hedgehog CoV obtained in this study and the DPP4 from different hosts, using Haddock 2.4. The values presented correspond to the mean and respective standard deviation of three replicates.

	DPP4					
	Human	Dog	Cat	Fox	Badger	Hedgehog
Hedgehog CoV	−78.2 ± 12.5	−86.5 ± 2.8	−94.2 ± 6.3	−95.9 ± 10.3	−90.3 ± 5.2	−78.7 ± 2.8
MERS-CoV	−154.8 ± 3.6	−128.5 ± 1.6	−130.3 ± 5.0	−130.5 ± 4.6	−138.1 ± 4.1	−131.7 ± 7.6

**Fig. 7:**
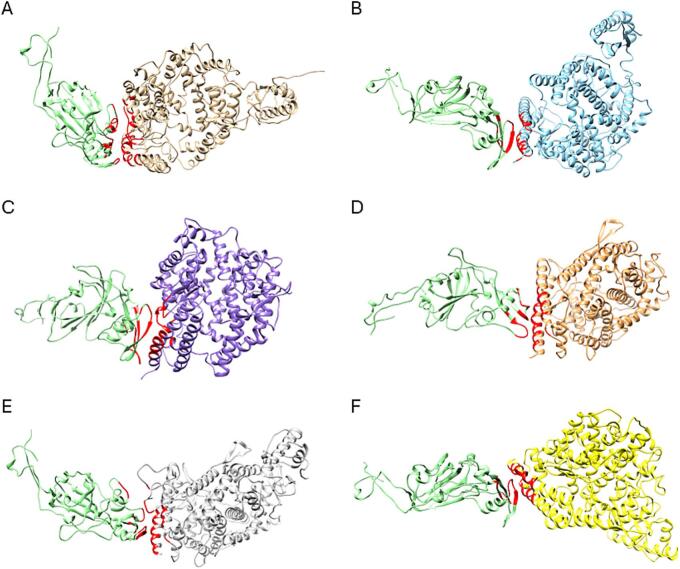
Models of structural interaction between the hedgehog CoV RBD (light green) and **(A)** human (beige), **(B)** dog (blue), **(C)** cat (purple), **(D)** fox (orange), **(E)** badger (grey) and **(F)** hedgehog (yellow) ACE2. The hedgehog CoV RBD residues that are within 5 Å of the contact surface with ACE2 are shown in red. (For interpretation of the references to colour in this figure legend, the reader is referred to the web version of this article.)

Considering the relatively low HADDOCK scores obtained for the interaction between the spike protein of hedgehog CoV and the DPP4 receptor of hedgehogs, it was hypothesized that hedgehog CoV might use an alternative receptor on the cell surface of hedgehogs. As close relatives of MERS-CoV in bats use ACE2 as their functional receptors, the binding interactions between hedgehog CoV spike and ACE2 from the same animal species were also tested. Protein docking simulations showed that the binding of the hedgehog CoV spike protein was strongest with human ACE2, followed by hedgehog ACE2, then with cat, fox and badger ACE2 and weakest with dog ACE2 (Fig. 7, Table 5).

**Table 5:** HADDOCK scores obtained in the protein docking studies between the RBD of spike protein from hedgehog CoV obtained in this study and the ACE2 from different hosts, using Haddock 2.4. The values presented correspond to the mean and respective standard deviation of three replicates.

	ACE2					
	Human	Dog	Cat	Fox	Badger	Hedgehog
Hedgehog CoV	−120.8 ± 3.8	−78.1 ± 2.1	−86.6 ± 4.3	−86.6 ± 9.7	−84.8 ± 4.4	−90.6 ± 5.5

## 4 Discussion

Hedgehogs have been suggested as potential natural reservoirs of coronaviruses in Europe and Asia [13,16,46]. Thus, this study aimed to assess and characterize the presence of coronaviruses in hedgehogs from Portugal. Our findings confirm the presence of *Betacoronavirus* and represent the first report of *Alphacoronavirus* in European hedgehogs in the country, with an overall coronavirus occurrence rate of 24.5 %. Coronavirus-positive samples were identified in both 2022 and 2023 sampling periods.

In 2013, a novel betacoronavirus closely related to MERS-CoV and clade C bat coronaviruses was discovered in European hedgehogs at an animal shelter in northern Germany [11]. Among 248 fecal samples tested, 58.9 % were positive for the novel coronavirus, named *Erinaceus coronavirus* (EriCoV) [11]. Subsequently, several studies reported the occurrence of EriCoV in *E. europaeus* across Europe. The occurrence rate found in the present study is higher than that identified in Great Britain (10.8 %; 38/351), similar to that found in Poland (25 %; 10/40) but lower than those reported in France (50 %; 37/74), in northern (58.3 %; 14/24) and central Italy (44.1 %; 45/102) [[12], [13], [14],^46^,^47^]. Variations between studies may be attributed to several factors, including sample size, geographic location, sampling season, sample handling and extraction procedures, and the detection method employed [13,46].

The majority (25 out of 27) of positive samples identified here belonged to *Betacoronavirus* found in hedgehogs (EriCoV), closely related to MERS-CoV. Additionally, two sequences corresponded to *Alphacoronavirus*, similar to Miniopterus bat coronavirus HKU8 *(Alphacoronavirus miniopteri)*. To the best of the authors knowledge, this is the first report of *Alphacoronavirus* in these animals. The *RdRp* sequences of the alphacoronaviruses are identical to those found in bats from Portugal. Interestingly, the positive hedgehog samples were collected in December 2022 from hedgehogs found in the northern region of the country (Maia and Porto), while the bat samples were collected in June 2023 in the central region. A possible transmission from bats to hedgehogs cannot be ruled out, as *M. schreibersii* is a migratory species that can potentially cover a territory the size of Portugal [48]. Furthermore, the hedgehogs came from the same rehabilitation center. Although the animals were housed in individual cages, their cohabitation in the same room, handling procedures, and sample collection several days after arrival at the rehabilitation center do not allow us to rule out the possibility of transmission between hedgehogs within the center.

Of the five samples sent for next-generation RNA sequencing, only one had RNA of sufficient quality for sequencing. The other samples were degraded or had low RNA integrity, likely due to travelling conditions, which hindered successful sequencing. The viable coronavirus-positive sample yielded 65 % of the full-length genome, showing the closest homology to *B. erinacei* previously identified in a European hedgehog in the United Kingdom. Given the geographical barrier and the fact that hedgehogs do not travel long distances or migrate, the similarity between the genome obtained in the present study and those of other European countries can likely be attributed to the co-evolution of coronaviruses with hedgehogs [14].

The first and essential step of viral infections involves the recognition of host cell receptors by viruses [21]. It is predicted that the hedgehog CoV receptor in host cells is the same as for MERS-CoV, DPP4. The contact interface between the MERS-CoV RBD and DPP4 comprises two major binding patches: in the first patch, the C-terminal end of the long loop connecting the β6 and β7 strands contacts blade 4 of DPP4, while in the second patch, a slightly concave outer surface on the MERS-CoV RBD accommodates a linker region containing a short α-helix between blades 4 and 5 of DPP4 [42]. Comparing the structural model of the hedgehog CoV RBD derived from this study to the MERS-CoV's, the hedgehog CoV RBD lacks the short β6 strand and the β5-β6 linking loop. However, it maintains part of the concave surface and the C-terminal end of the long loop that, in MERS-CoV, connects the β6 and β7 strands. This structural similarity suggests a potential for interaction with DPP4.

To assess the risk of cross species transmission of the coronavirus partially sequenced in this study, the binding affinity between the hedgehog CoV spike RBD and the DPP4 of animal species that are known to interact with hedgehogs and may serve as a source of transmission to humans were analyzed through protein docking studies. Hedgehogs can occasionally be prey of red foxes and even killed by stray dogs [49]. The spines of hedgehogs deter most other predators except the Eurasian eagle owl (*Bubo bubo*) and the Eurasian badger (*Meles meles*) that possess the capability to regularly eat adult hedgehogs by turning them over and opening their abdomen with their claws [50]. In northern Europe, badgers are considered the primary predators of hedgehogs, although badger population densities are significantly lower in continental Europe [51]. On the other hand, cats and hedgehogs often have antagonistic encounters in urban gardens, possibly due to cats' hunting instinct or simply their curiosity, although they almost always emerge submissive, likely due to the hedgehogs' spines [52]. Thus, foxes, badgers, dogs and cats can act as intermediary species for potential transmission of pathogens from hedgehogs to humans. When comparing the interaction between the hedgehog CoV RBD and DPP4 of human, dog, cat, fox, badger and hedgehog, it was observed that the predicted binding of the hedgehog CoV RBD was strongest with DPP4 of fox and cat and weakest with hedgehog and human DPP4. Consequently, if there is a risk of cross-species transmission of the hedgehog CoV, it is predicted to be higher for foxes and cats. These docking predictions suggest that the interaction between the spike protein of hedgehog CoV and the hedgehog DPP4 is not very stable, raising the question of whether this interaction occurs *in vivo* or if hedgehog CoV relies on another receptor to enter host cells.

A study conducted in China, which involved sequence analysis and structural modeling of the spike protein from the *E. amurensis* hedgehog coronavirus HKU31, a close relative of the European hedgehog CoV, suggested that this hedgehog CoV is unlikely to bind to human DPP4 [16]. The same conclusion was drawn for Italian EriCoV based on sequence and structure analysis of the spike protein [15]. These findings raise questions about which receptor hedgehog CoV might use to enter host cells. The carcinoembryonic antigen-related cell adhesion molecule 5 (CEACAM5) has been reported as a cell surface binding target of MERS-CoV, enhancing the virus's attachment to the host cell surface, thereby facilitating MERS-CoV infection [53]. However, to our knowledge, this protein has not been described in hedgehogs. On the other hand, NeoCoV (a *Merbecovirus* isolated from *Neoromicia capensis*, the closest known MERS-CoV relative found in bats, and its close relative PDF-2180 isolated from *Pipistrellus hesperidus* utilize ACE2 for efficient cellular entry [23]. In the same study, the phylogenetic analysis based on the amino acid sequences of the spike protein revealed a distant evolutionary relationship with MERS-CoV but a close relationship with EriCoVs. In this context, the interaction between the hedgehog CoV RBD and the ACE2 of hedgehogs and other animal species that may interact with them was tested. Surprisingly, the hedgehog CoV RBD exhibited the strongest predicted binding with human ACE2, and its interaction with hedgehog ACE2 was stronger than with hedgehog DPP4. It's crucial to acknowledge that these are *in silico* predictions considering only the spike protein and the adaptation of other viral proteins has not been considered. However, these results suggest that hedgehog CoV could use a different receptor than MERS-CoV, namely ACE2, to enter host cells, and that the risk of cross-species transmission is higher to humans.

A study conducted in 2016 performed a phylogenetic analysis of the whole genomes of viruses related to MERS-CoV [54]. They inferred that the ancient MERS-CoV has existed for decades, suggesting that the ancestral MERS-CoV may have infected multiple animal hosts, such as bats or hedgehogs, over this period, and only recently gained the ability to infect humans or camels. In the same phylogenetic analysis, NeoCoV, found to use ACE2 for efficient cellular entry [23], emerged as a more recent ancestor of MERS-CoV than EriCoV, supporting the hypothesis that ancestors of MERS-CoV might have utilized different host cell receptors. Regarding SARS-CoV and MERS-CoV transmission to humans, it has been suggested that different receptor binding motifs from bat viruses that are capable of infecting humans were obtained during crossover events, possibly through recombination events, with non-specific RBD motifs obtained in this process [55,56].

This study contributes to a deeper understanding of the epidemiology and ecology of coronaviruses in hedgehogs from Portugal. The current findings highlight the urgent need for more concrete and comprehensive investigations. It is crucial to assess the actual binding capacity of these coronaviruses *in vitro*. The uncertainty surrounding its interaction with known receptors suggests the possibility of an alternative entry mechanism, emphasizing the need for further research to identify the specific receptor involved in the viral entry process. Additionally, it would be valuable to investigate the potential occurrence of these viruses in other small carnivores, such as shrews, and to study the transmission dynamics in bats and hedgehogs in order to determine whether the presence of alphacoronaviruses are isolated spillover events or if the virus is established in hedgehog or bat populations. These lines of research are essential for evaluating the actual risk of cross-species transmission, thereby aiding in the assessment and mitigation of potential public health risks. The present study underscores the importance of continuous monitoring and the need for more robust follow-up investigations to address these critical questions.

## 5 Conclusion

In the present study, the presence of *Betacoronavirus* in *E. europaeus* from Portugal is described, along with the first report of *Alphacoronavirus* in this species. The detection of *Alphacoronavirus* sequences identical to those found in Portuguese bats requires further investigation to understand possible interspecies transmission. The RNA sequencing results of a *Betacoronavirus* and phylogenetic analysis further indicate a co-evolutionary relationship between hedgehog coronaviruses and their hosts across Europe. Additionally, protein docking studies raise the question of whether hedgehog CoV utilizes the same receptor as MERS-CoV to enter host cells and highlight the potential risk of cross-species transmission of EriCoV, particularly to humans. This study enhances our understanding of coronavirus epidemiology in hedgehogs, emphasizing the urgent need for further investigations into cross-species transmission. Ongoing surveillance and comprehensive studies are essential to address the potential public health threats posed by these viruses.

## CRediT authorship contribution statement

**Andreia V.S. Cruz:** Writing – original draft, Methodology, Investigation, Formal analysis, Data curation, Conceptualization. **Sérgio Santos-Silva:** Writing – review & editing, Investigation, Data curation. **Luís Queirós-Reis:** Writing – review & editing, Methodology, Formal analysis. **Clarisse Rodrigues:** Resources, Data curation. **Vanessa Soeiro:** Resources, Data curation. **Rachael E. Tarlinton:** Writing – review & editing, Supervision, Project administration, Methodology, Funding acquisition, Formal analysis. **João R. Mesquita:** Writing – review & editing, Supervision, Project administration, Methodology, Funding acquisition, Conceptualization.

## Declaration of competing interest

The authors declare no conflicts of interest related to this work.

## Data Availability

All partial *RdRp* sequences obtained in this study are available in GenBank under the accession numbers OQ703947–OQ703961, OQ714708, and OR506455–OR506458, and the partial genome is available under the accession number PP724614.
